# Presence and Characterization of a Novel *cfr*-Carrying Tn*558* Transposon Derivative in *Staphylococcus delphini* Isolated From Retail Food

**DOI:** 10.3389/fmicb.2020.598990

**Published:** 2021-01-15

**Authors:** Feng Zhang, Shi Wu, Jiahui Huang, Runshi Yang, Jumei Zhang, Tao Lei, Jingsha Dai, Yu Ding, Liang Xue, Juan Wang, Moutong Chen, Qingping Wu

**Affiliations:** ^1^School of Bioscience and Bioengineering, South China University of Technology, Guangzhou, China; ^2^Guangdong Provincial Key Laboratory of Microbial Safety and Health, State Key Laboratory of Applied Microbiology Southern China, Guangdong Institute of Microbiology, Guangdong Academy of Sciences, Guangzhou, China; ^3^Department of Food Science and Technology, Jinan University, Guangzhou, China; ^4^College of Food Science, South China Agricultural University, Guangzhou, China

**Keywords:** Tn*558*, *cfr*, *Staphylococcus delphini*, unconventional circularizable structure, multidrug resistance

## Abstract

Antimicrobial resistance has become a major public health threat. Food-related *Staphylococcus* species have received much attention due to their multidrug resistance. The *cfr* gene associated with multidrug resistance has been consistently detected in food-derived *Staphylococcus* species. In this retrospective study, we examined the prevalence of *cfr*-positive *Staphylococcus* strains isolated from poultry meat in different geographical areas of China from 2011 to 2016. Two *cfr*-positive *Staphylococcus delphini* strains were identified from poultry meat in China. Comparative and whole-genome analyses were performed to characterize the genetic features and overall antimicrobial resistance genes in the two *S. delphini* isolates 245-1 and 2794-1. Whole-genome sequencing showed that they both harbored a novel 20,258-bp *cfr*-carrying Tn*558* transposon derivative on their chromosomes. The Tn*558* derivative harbors multiple antimicrobial resistance genes, including the transferable multiresistance gene *cfr*, chloramphenicol resistance gene *fexA*, aminoglycoside resistance genes *aacA-aphD* and *aadD*, and bleomycin resistance gene *ble*. Surprisingly, within the Tn*558* derivative, an active unconventional circularizable structure containing various resistance genes and a copy of a direct repeat sequence was identified by two-step PCR. Furthermore, core genome phylogenetic analysis revealed that the *cfr*-positive *S. delphini* strains were most closely related to *S. delphini* 14S03313-1 isolated from Japan in 2017 and 14S03319-1 isolated from Switzerland in 2017. This study is the first report of *S. delphini* harboring a novel *cfr*-carrying Tn558 derivative isolated from retail food. This finding raises further concerns regarding the potential threat to food safety and public health safety. The occurrence and dissemination of similar *cfr*-carrying transposons from diverse *Staphylococcus* species need further surveillance.

## Introduction

In recent years, resistance in bacteria has spread worldwide and presents a serious threat to human health. Linezolid is an oxazolidinone antibiotic and is considered as the last-resort antibiotic for the treatment of infections caused by multidrug-resistant (MDR) Gram-positive pathogens, including *Staphylococcus* species (Wilson et al., 2008). The antibiotic targets the P site in the peptidyl transferase center of the 23S ribosomal RNA of the 50S ribosomal subunit, acting on this target and blocking protein synthesis (Aoki et al., 2002). In fact, due to the synthetic nature of the drug, resistance to this antibiotic is rare. However, the *cfr* gene could mediate resistance to linezolid (Long et al., 2006). This gene encodes a methyltransferase that catalyzes the posttranscriptional methylation of adenosine at nucleotide position 2503 (*Escherichia coli* numbering) in 23S rRNA, which replaced the target of binding for linezolid (Corinna et al., 2005; Giessing et al., 2009; Anna et al., 2016). However, due to overlapping binding sites, *cfr* methylation also confers resistance to four other classes of antimicrobial agents and results in the PhLOPSA multiresistance phenotype, including resistance to phenicols, lincosamides, oxazolidinones, pleuromutilins, and streptogramin A compounds (Long et al., 2006; Anna et al., 2016). Interestingly, *cfr* is often associated with *erm*, *fexA*, *lsa*(B), and *tet*(L), which can assist in co-selecting the *cfr* gene and in its spread (Shen et al., 2013; Mendes et al., 2014).

Generally, the *cfr* gene is often associated with mobile genetic elements (MGEs) (plasmids, integrative, and conjugative elements or transposons), which have great potential for dissemination (Shen et al., 2013). Tn*558* is one of these bacterial transposons and was first identified on the plasmid pSCFS2 harboring the antimicrobial resistance gene (ARG) *fexA* from *Staphylococcus lentus* (Kehrenberg and Schwarz, 2005). Currently, this transposon is often harbored with *cfr*, and derivatives of Tn*558* usually carry other acquired ARGs (Kehrenberg et al., 2007; Li et al., 2018). Therefore, this transposon plays an important role as vectors in the spread of transposon-borne ARGs.

Members of the genus *Staphylococcus* are widespread in nature and play vital roles in disease causation in humans and animals (McGavin and Heinrichs, 2012; Vrbovská et al., 2020). Among these species, *Staphylococcus delphini* is a pathogen that causes animal and human infections (Magleby et al., 2019; Ruiz-Ripa et al., 2019). It belongs to the *Staphylococcus intermedius* group and was first described in purulent skin lesions of dolphins (Varaldo et al., 1988). *S. delphini* is further separated into two subgroups, groups A and B, based on the phylogenetic analysis of the *sodA*, *hsp60*, and *nuc* genes and DNA–DNA hybridization (Sasaki et al., 2007). Although this staphylococcal species is poorly documented due to misidentification with *S. intermedius*, it has been isolated from humans and a wide range of diseased animals, including domestic pigeons, camels, horses, magpies, cinereous vultures, and mustelids, which serve as the natural hosts of *S. delphini* group A (Devriese et al., 2005; Sasaki et al., 2007; Sledge et al., 2010; Guardabassi et al., 2012; Sudagidan and Aydin, 2012; Stull et al., 2014; Magleby et al., 2019; Ruiz-Ripa et al., 2019).

In this retrospective study, we examined the prevalence of *cfr*-positive *Staphylococcus* isolates in poultry meat from 2011 to 2016. We determined the complete genome sequence of *cfr*-positive *S. delphini* and described their phenotypic and genotypic profiles. This is the first report of a Tn*558* derivative-embedded *cfr* in *S. delphini* isolated from retail food.

## Materials and Methods

### Bacterial Isolation

From July 2011 to June 2016, we collected 4,300 retail food samples from supermarkets, fairs, and farmer markets, covering most of the provincial capitals of China (Supplementary Figure 1), and isolated 1,581 *Staphylococcus* strains, including *Staphylococcus aureus*, *Staphylococcus argenteus*, *S. delphini*, *Staphylococcus epidermidis*, and other staphylococci from 1,063 positive samples from all the sampling sites (Wu et al., 2018a,b). During the retrospective study of *cfr*-positive *Staphylococcus* species among these isolates, the *cfr*-positive strains 245-1 and 2794-1 were isolated from frozen duck wings in Guangzhou 2013 and frozen duck legs in Kunming 2014, respectively. The isolates were further identified as *S. delphini* by the MALDI-TOF/MS system (Bruker, Bremen, Germany) (Decristophoris et al., 2011).

### PCR Detection

The presence of the resistance gene *cfr* was identified by PCR and Sanger sequencing (Kehrenberg et al., 2009). The presence of the two direct repeats (DRs) and circular intermediate translocatable units (TUs) was detected by PCR and inverse PCR (the primers and conditions are shown in Table 2). To minimize the detection of artificial products, a high-fidelity polymerase (PrimeSTAR GXL DNA Polymerase, Takara, Dalian, China) and an 8-min elongation step were used (Tansirichaiya et al., 2016). The amplicons obtained by PCR and inverse PCR experiments were subjected to Sanger sequencing.

### Antimicrobial Susceptibility Testing

Minimum inhibitory concentrations (MICs) were determined using a standard broth dilution method according to the CLSI guidelines with *S. aureus* ATCC 29213 as a quality control strain (Weinstein and Clinical and Laboratory Standards Institute, 2018). The MICs for all of the following antimicrobials were determined: FFC, florfenicol; CHL, chloramphenicol; CLI, clindamycin; TIA, tiamulin; LZD, linezolid; K, kanamycin; ERY, erythromycin; FOX, cefoxitin; VAN, vancomycin; RIP, rifampicin; and DAP, daptomycin. The MIC breakpoints of each antibiotic, except florfenicol, were used as recommended by the current CLSI guidance (Weinstein and Clinical and Laboratory Standards Institute, 2018). For florfenicol, the results were interpreted according to the Veterinary CLSI (VET01-A5).

### Whole-Genome Sequence and Analysis

Genomic DNA for whole-genome sequencing was extracted from the *cfr*-positive strains using a genomic extraction kit (Magen Biotech, Guangzhou, China) according to the manufacturer’s instructions. Whole-genome sequencing of the *cfr*-positive strains was performed using the Illumina HiSeq Xten platform (800-bp paired-end reads with 100-fold average coverage) and a PacBio Sequel II sequencing instrument (100-fold average read depth). The chromosome sequences were assembled into one scaffold using the software SMRT Portal, version 3.2.0. The genomic DNA annotation was performed in Prokka NCBI-BLASTP/BLASTX (Torsten, 2014). The single-nucleotide polymorphisms (SNPs) between strains 245-1 and 2794-1 were identified with Snippy software^1^.

The acquired antibiotic resistance genes were identified by ResFinder 3.0^2^ and were further verified through a BLAST search against the Comprehensive Antibiotic Resistance Database (Ea et al., 2012). The genetic environment of the *cfr* gene was analyzed using BLAST^3^, followed by visualization of the comparative *cfr* multiresistance region (MRR) with Easyfig, v2.2.2 (Sullivan et al., 2011).

### Phylogenetic Analysis

All publicly available draft genome sequences of *S. delphini* strains were acquired (22 strains with at least 50 × read coverage), and core SNP alignments were produced *via* Snippy using the *S. delphini* 8086 complete genome sequence (ASM30811v1) as a reference (see text footnote 1). The maximum-likelihood (ML) phylogenetic tree was constructed with RAxML-NG based on the ML optimality criterion (Kozlov et al., 2019). The locations of recombined regions on each branch were detected, and this tree was reconstructed by ClonalFrameML (Didelot and Wilson, 2015). FigTree, v1.4.3, was used to finalize the tree visualization (Morariu et al., 2008).

### Nucleotide Sequence Accession Numbers

The complete genomic sequences of 245-1 and 2794-1 have been deposited in GenBank: 245-1 (GenBank ID: CP063368) and 2794-1 (GenBank ID: CP063367).

## Results

### Phenotypic Characteristics of *cfr*-Positive *S. delphini*

In this study, 245-1 and 2794-1 displayed the same MDR profiles. Antimicrobial susceptibility testing showed that these strains were resistant to chloramphenicol, florfenicol, tiamulin, clindamycin, and linezolid, exhibiting a high level of resistance to florfenicol (MIC = 256 μg/ml), chloramphenicol (MIC > 128 μg/ml), and tiamulin (MIC > 128 μg/ml). Moreover, the isolates were susceptible to vancomycin, daptomycin, and rifampicin (Table 1).

**TABLE 1:** Phenotypic and genotypic characteristics of *Staphylococcus delphini*.

**Bacterial isolate**		**MIC (μg/mL)**										**Resistance genes**
	**FFC**	**CHL**	**CLI**	**TIA**	**LZD**	**K**	**ERY**	**FOX**	**VAN**	**RFP**	**DAP**	
245-1	256	128	4	128	8	16	0.25	0.5	1	<0.015	0.25	*cfr*, *fexA*, *ble*, *aacA-aphD*, *aadD*
2794-1	256	128	4	128	8	16	0.25	0.5	1	<0.015	0.5	*cfr*, *fexA*, *ble*, *aacA-aphD*, *aadD*
29213	8	8	0.0625	0.25	4	1	0.125	4	0.5	0.0078	0.5	NONE

*FFC, florfenicol; CHL, chloramphenicol; CLI, clindamycin; TIA, tiamulin; LZD, linezolid; K, kanamycin; ERY, erythromycin; FOX, cefoxitin; VAN, vancomycin; RIP, rifampicin; DAP, daptomycin.*

### Basic Genomic Information for *cfr*-Positive *S. delphini*

**FIGURE 1:**
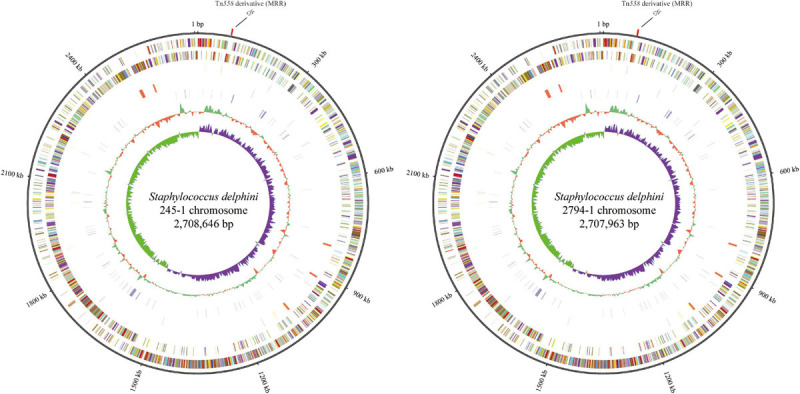
Circular representation of the *cfr*-positive *Staphylococcus delphini* 245-1 and 2794-1 genomes. From the outer to the inner circles in the chromosome circular map: slot 1 (ARGs) and slots 2–9 (slot 2, genome size; slot 3, forward strand gene, colored according to the cluster of orthologous groups classification; slot 4, reverse strand gene, colored according to the cluster of orthologous groups classification; slot 5, forward strand ncRNA; slot 6, reverse strand ncRNA; slot 7, repeat; slot 8, GC content; and slot 9, GC skew).

To understand the molecular characteristics and resistomes of the two strains of *S*. *delphini*, they were submitted for whole-genome sequencing. Basic information related to the complete genome sequence of *cfr*-positive *S. delphini* is shown in Figure 1. The chromosomes of 245-1 and 2794-1 consisted of 2,708,646 bp with 2,486 predicted ORFs along with 102 RNAs and 2,707,963 bp with 2,486 predicted ORFs along with 102 RNAs, respectively. The genome analysis of the complete chromosomal DNA revealed that there were 166 variants between the chromosomes of 245-1 and 2794-1, and there were multiple ARGs located on their chromosomes, including *fexA* (conferring resistance to chloramphenicol), *aacA-aphD* and *aadD* (resistance to aminoglycosides), *ble* (resistance to bleomycin), and the multiresistance gene *cfr* (resistance to phenicols, lincosamides, oxazolidinones, pleuromutilins, and streptogramin A).

### Core Genome Phylogenetic Analysis of *cfr*-Positive *S. delphini*

**FIGURE 2:**
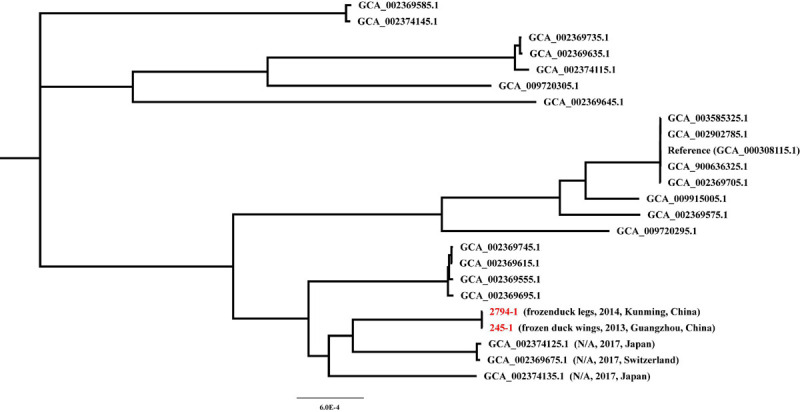
Maximum-likelihood (ML) core genome phylogeny of *cfr*-positive *Staphylococcus delphini* 245-1 and 2794-1 based on the ML method.

To further investigate the potential sources of *cfr*-positive *S. delphini* 245-1 and 2794-1, we performed a core genome phylogenetic analysis of all publicly available draft genome sequences of *S. delphini* strains. The phylogenetic analysis shows that 245-1 and 2794-1 are most closely related to *S. delphini* 14S03313-1 (GCA_002374125.1) isolated from Japan in 2017 and 14S03319-1 (GCA_002369675.1) isolated from Switzerland in 2017 (Figure 2). This phylogenetic analysis did not reveal the origin of 245-1 and 2794-1, indicating that the scarcity of genomic sequences may be the constraint, and further genomic sequencing is needed to identify the source of the *cfr*-positive strains.

### Genetic Environment of *cfr* Located on a Novel Tn*558* Transposon Derivative

**FIGURE 3:**
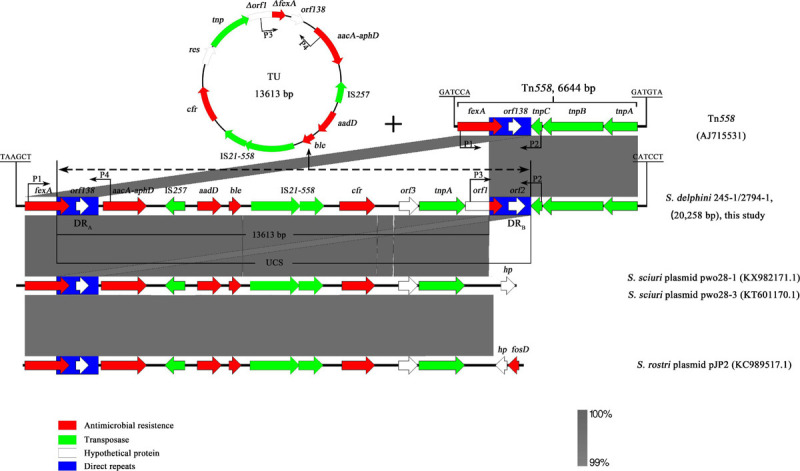
Schematic presentation of the environment of the *cfr* gene on chromosomes 245-1 and 2794-1 and the formation of translocatable units (TUs) mediated by direct repeats (DRs). The TUs derived from the region between DR_A_ and DR_B_ and the remaining structures after the excision of unconventional circularizable structures on chromosomes. Areas shaded in gray indicate homologous regions of ≥99% nucleotide sequence identity. Arrows indicate the orientations of the open reading frames, and the colors are based on their predicted gene functions. Frames with blue represent DRs. “*Delta*” represents a truncated gene. The figure is drawn to scale.

Genomic mining revealed that the *cfr* gene, along with four other ARGs, namely, *fexA*, *aacA-aphD*, *aadD*, and *ble*, was located on a 20,258-bp (62,847–83,104 nt on the chromosomes of 245-1 and 2794-1 in Figure 1) MRR on the chromosomes. Further BLAST analysis showed that the ARGs *aacA-aphD*, *aadD*, *ble*, and *cfr* were flanked by two DRs oriented in the same direction within the MRR and that the two DRs both belonged to Tn*558* (Figure 3). The presence of the two DRs was further identified by PCR assays followed by sequencing of the amplicons (primers shown in Table 2). Both DRs were 1,326 bp in size, except for 18-bp exchanges in DR_B_ compared to DR_A_. DR_A_ contained partial *fexA* (430 bp) and *orf138* sequences, while DR_B_ comprised partial *orf1* (430 bp) and *orf2* sequences. Further analysis revealed that the single-nucleotide exchange TAG (*orf138*) → TAC (*orf2*) caused the termination codon to mutate to a Tyr codon, resulting in an extension of the open reading frame that transformed *orf138* to *orf2*.

**TABLE 2:** Primers used for detecting antibiotics resistance genes, the circular forms and the structures not included in the corresponding region of the unconventional circularizable structures.

**Primer**	**Sequence (5′ to 3′)**	**Product size (bp)**	**Annealing temperature (°C)**	**Purpose**
Tn-F	CGGTGCCTAATCATTCGTATGC	872	55	Detection of minicircle form of Tn*558*
Tn-R	CGCTTAACCGGTTCTATGACTTCA			
P1	GAAAAACGGTTGGCACGGTA	1824	65	Detection of the formation of translocatable units (TUs) between DR_A_ and DR_B_
P2	CTTCATCTTCCCAAGGCTCTGT			
P3	GGCAGAATCCGTAGGAAGCA	1817	65	Detection of remaining structures after the excision of UCSs on chromosomes
P4	CCCTCGTTCAGAGGACGTAT			
A-F	TCGTCCCATTGCTAGTCGTT	1683	55	Detection of DR_A_
A-R	AAAACTTCATCTTCCCAAGGCT			
B-F	TGCCTGGAATCGAAAAACGG	1680	55	Detection of DR_B_
B-R	CCCTCGTTCAGAGGACGTATT			
cfr-F	TGAAGTATAAAGCAGGTTGGGAGTCA	746	58	Detection of *cfr*
cfr-R	ACCATATAATTGACCACAAGCAGC			

*Primers Tn-F/Tn-R were used for detecting the formation of minicircle of Tn*558* derivative; primers P1/P2 were used for detecting the formation of translocatable units (TUs) between DR_A_ and DR_B_; primers P3/P4 were used for detecting the remaining structures after the excision of UCSs on chromosomes; primers A-F/A-R, B-F/B-R were used for detecting DR_A_ and DR_B_; primers cfr-F/cfr-R were used for detecting resistance gene *cfr*.*

To further determine whether these unknown DRs in 245-1 and 2794-1 could mediate the formation of circular intermediate TUs, inverse PCR (P3, P4) was performed, followed by sequencing of the amplicons. Two identical PCR products (1,824 bp) were acquired from 245-1 and 2794-1, including a copy of DR_B_, *orf138*, and part of *fexA*, as determined by sequencing (Figure 3). The TUs (13,613 bp) resulted from the recombination between DR_A_ and DR_B_, including multi-ARGs and one copy of DR_A_. The PCRs (P1, P2) containing one copy of DR_B_ detected the remaining structures after the excision of unconventional circularizable structures (UCSs) on chromosomes, and the results were consistent with the inverse PCR results (Figure 3). Importantly, the remaining structures were Tn*558*. These results confirmed the excision and cyclization of the structure (Figure 3). Further BLAST analysis revealed that the left Δ*fexA*-UCS exhibited 99.88% nucleotide identity to the corresponding region of the plasmids pWo28-1 (KX982171.1) and pWo28-3 (KY601170.1) from *Staphylococcus sciuri* and the plasmid pJP2 (KC989517.1) from *Staphylococcus rostri* lacking DR_B_ (Figure 3).

The sequence alignment analysis showed that the *cfr* MRR consisted of a Tn*558* homologous region (6,644 bp) and a 13,613-bp region (Figure 3). This arrangement is a novel derivative of the Tn*558* transposon. Compared to the *fexA*, *orf138*, *tnpC*, *tnpB*, and *tnpA* genes in Tn*558* (Kehrenberg and Schwarz, 2005), a closer inspection of the Tn*558* derivative showed that several nucleotide exchanges were identified in *fexA* (14 bp), *tnpB* (20 bp), and *tnpA* (14 bp), except for *orf138*. To further explain the genetic environment of the *cfr* MRR in this study, the plasmids pWo28-1 (KX982171.1) and pWo28-3 (KY601170.1) from *S. sciuri* and plasmid pJP2 (KC989517.1) from *S. rostri* are also shown in Figure 3. Analysis of the regions flanking the Tn*558* derivative insertion in the chromosome identified a reading frame encoding a putative protein of 114 aa (62,844–62,846 and 83,105-83,443 nt on chromosomes 245-1 and 2794-1) that shared 98.54% nucleotide identity with a 148-aa DNA repair protein from *Macrococcus canis* (CP021059.1) (Gobeli et al., 2017). Additionally, a minicircle of Tn*558*, an indication of Tn*558* having transposition activity, was identified *via* PCR (primers shown in Table 2) and sequencing of the derivative.

## Discussion

Naturally, *S. delphini* is widely susceptible to clinically relevant classes of antibiotics. In a previous study from Denmark, among 55 *S. delphini* isolates recovered from mink, only some isolates were resistant to tetracycline (51%), penicillin (47%), and erythromycin (20%), whereas all the isolates tested susceptible to a vast majority of the antimicrobials assayed, including cefoxitin (Nikolaisen et al., 2017). In 2019, Magleby et al. also reported the first human case of *S. delphini* infection and found that the isolate exhibited low MIC values for all the antimicrobials assayed, including oxacillin (Magleby et al., 2019). Remarkably, the multiresistance gene *cfr* was shown to encode Cfr, an RNA methyltransferase that affects the binding of at least five chemically unrelated antimicrobial classes, namely, phenicols, lincosamides, oxazolidinones, pleuromutilins, and streptogramin A antibiotics, ultimately leading to a multidrug resistance phenotype (Long et al., 2006). Thus, the emergence and the global spread of the multiresistance gene *cfr* reduce the efficacy of a number of antibiotics in the control of Gram-positive bacteria. In this study, we identified the *cfr* gene in two food-related *S. delphini* strains. To the best of our knowledge, this study is the first report of the *cfr* gene existing in *S. delphini*. Furthermore, the *cfr* gene was located in an MRR with a number of antibiotic genes (*fexA*, *aacA-aphD*, *aadD*, and *ble*). The coexistence of *cfr* and other ARGs limits the choice of antibiotic therapy and may lead to the co-selection of these genes even without direct selection pressure, thereby increasing the retention and dissemination of these ARGs in *Staphylococcus.*

In this study, MRRs, including *cfr* and other ARGs, were confirmed as novel derivatives of the Tn*558* transposon. Tn*558* is a 6.6-kb bacterial transposon. It was first identified on the plasmid pSCFS2 harboring ARG *fexA* from *S. lentus*, and then numerous derivatives harboring numerous ARGs were found (Kehrenberg and Schwarz, 2005; Kehrenberg et al., 2007; Li et al., 2018). With a few exceptions, *cfr* is often harbored in the Tn558 transposon as coexisting with other ARGs, such as *fexA*, *mecA*, *erm*(A/B/C), *tet*(K/L/M), and *drf*(K/G) in the plasmids pSCFS3, pSCFS6, and pSCFS7 in previous studies (Witte and Cuny, 2011), but in this study, the derivative of the Tn*558* transposon harbored *cfr*, *fexA*, *aacA-aphD*, *aadD*, and *ble* on the chromosomes. In addition, the additional DRs within the Tn*558* derivative further confirm the particularity of this transposon. As previously reported for Tn*558* derivatives, there are no inverted repeats at the ends and no duplication of the target sequence at the integration site of the Tn*558* derivative. The typical 6-bp core sequences 5′-GATGTA-3′ at the left-end junction and 5′-GATCCA-3′ at the right-end junction were replaced by 5′-CATCCT-3′ and 5′-TAAGCT-3′ in the novel derivative. The disappearance of target duplication and the alteration of the typical core sequences may have occurred during the transposition process (Diaz-Aroca et al., 1987; Murphy, 1990). Moreover, the reading frame, including the insertion site of the Tn*558* derivative, is similar to the protein containing the Tn*558* site, and the excision of TUs in this Tn*558* derivative could lead to the formation of Tn*558*, indicating that the DR_A_ and DR_B_ in this study may be involved in the evolution of Tn*558* and that this derivative may be the ancestor of Tn*558* (Kehrenberg and Schwarz, 2005). Although multiple conjugation assays failed, the presence of a circular Tn*558* structure is indicative of the functional activity, suggesting that this novel Tn*558* derivative is a transposable element and may mediate the transfer of the *cfr* gene in the process of transposition (Kehrenberg and Schwarz, 2005).

Generally, the *cfr* gene often coexists with other ARGs on transposons or plasmids and is often in close proximity to insertion sequences (ISs), such as IS*21-558*, IS*256*, or IS*Enfa4*, which play a crucial role in the mobility of *cfr* (Witte and Cuny, 2011; Wendlandt et al., 2015). These mobile structures have been detected among several Gram-positive bacteria, such as staphylococci, *Enterococcus faecalis*, *Macrococcus caseolyticus*, *Jeotgalicoccus pinnipedialis*, *Bacillus* spp., and *Streptococcus suis*, as well as in Gram-negative bacteria, such as *E. coli* and *Proteus vulgaris* (Shen et al., 2013). However, mobile structures can form UCSs (Palmieri et al., 2013). UCSs lack recombinase genes and can be excised in circular form due to the extensive DRs flanking the DNA segment undergoing excision (Locke et al., 2012; Palmieri et al., 2012, 2013). Thus, they are very important for the horizontal transmission of ARGs. In this study, the ARGs *aacA-aphD*, *aadD*, *ble*, and *cfr*, bracketed by DRs, formed a novel genetically mobile structure. The particular genetic structures identified by the analysis were referred to as UCSs. Two-step PCR results indicated that this structure can be looped out and excised from the chromosome, leading to the formation of Tn*558* (Figure 3), which suggests that the DR is active and involved in the mobility of the Tn*558*-carried *cfr* gene in this study. Further BLAST analysis revealed that the left Δ*fexA*-UCS exhibited 99.88% nucleotide identity to the corresponding region of the plasmids pWo28-1 (KX982171.1) and pWo28-3 (KY601170.1) from *S. sciuri* and plasmid pJP2 (KC989517.1) from *S. rostri* lacking DR_B_ (Figure 3). Therefore, the DR_A_ and DR_B_ in this study, similar to ISs, might facilitate the dissemination and accumulation of ARGs in Tn*558* (Palmieri et al., 2013; Harmer et al., 2014). Of course, the functions of these two unknown DRs still need to be further studied and explored in the future.

Unconventional circularizable structures are widely distributed in Gram-negative and Gram-positive bacteria and play an important role in the dissemination of ARGs (Palmieri et al., 2013; Chanchaithong et al., 2019). The DRs in UCSs are usually long and are more than 100 times longer than the *att* sites functioning in traditional MGEs (Frost et al., 2005). The DRs may contain genes, such as *erm(B)*, *mef*, (macrolide efflux), and *ofr138* in this study, but they are not involved in transposition (Locke et al., 2012; Hao et al., 2019). The exact mechanism of mobilization has not been determined, although hypotheses have been proposed (Azpiroz et al., 2011). This transfer mechanism may be similar to that of IS*26 via* site-specific recombination, including a multistep process that requires the formation of a TU, precise excision of the TU, and integration targeting the preexisting DR (Harmer et al., 2014; Harmer and Hall, 2015). The endogenous instability of UCSs endows the encompassed niche adaptation determinants with the ability to be transferred. Moreover, they are often carried by MGEs, which prompts the updating of MGEs (such as the derivative of Tn*558*) and further accelerates the spread of UCSs. Furthermore, the presence of DRs on this novel *cfr*-carrying Tn*558* derivative may accelerate the spread and persistence of ARGs among staphylococci and exacerbate the threat of superbugs, such as methicillin-resistant *S. aureus*. The proliferation of the transferable ARG *cfr* kidnapped by transposons or other MGEs has impaired the efficiency of oxazolidiones in clinical settings and threatens public health (Li et al., 2018).

## Conclusion

To the best of our knowledge, this study is the first report of *S. delphini* harboring a novel *cfr*-carrying Tn*558* derivative. The constant occurrence of the *cfr* gene in new staphylococcal host species underlines its strong transmissibility and wide distribution. This finding raises further concerns regarding the potential threat to food safety and public health safety. The occurrence and the dissemination of similar *cfr*-carrying transposons from diverse *Staphylococcus* species need further surveillance.

## Data Availability Statement

The complete genomic sequences of 245-1 and 2794-1 have been deposited in GenBank: 245-1 (GenBank ID: CP063368) and 2794-1 (GenBank ID: CP063367).

## Author Contributions

QW, JZ, SW, and TL conceived and designed the experiments. FZ, JH, and JD performed the experiments. FZ, SW, and RY analyzed the data. YD, LX, MC, and JW contributed reagents, materials, and analysis tools. FZ, SW, and JW contributed to the writing of the manuscript. All authors contributed to the article and approved the submitted version.

## Conflict of Interest

The authors declare that the research was conducted in the absence of any commercial or financial relationships that could be construed as a potential conflict of interest. The reviewer JS declared a shared affiliation with one of the authors JW to the handling editor at the time of review.

## Footnotes

1 https://github.com/tseemann/snippy

2 https://cge.cbs.dtu.dk/services/ResFinder/

3 http://blast.ncbi.nlm.nih.gov/Blast.cgi

## Supplementary Material

Supplementary Figure 1 Sample collection locations for this study in China.
