# Supplementary material for: Presence and Characterization of a Novel cfr-Carrying Tn558 Transposon Derivative in Staphylococcus delphini Isolated From Retail Food
Source: Front Microbiol. 2021 Jan 15;11:598990. doi: 10.3389/fmicb.2020.598990 (PMC7843796; doi:10.3389/fmicb.2020.598990)
Supplement: Supplementary Figure 1 — Sample collection locations for this study in China. [file Image_1.pdf]

Supplementary Material

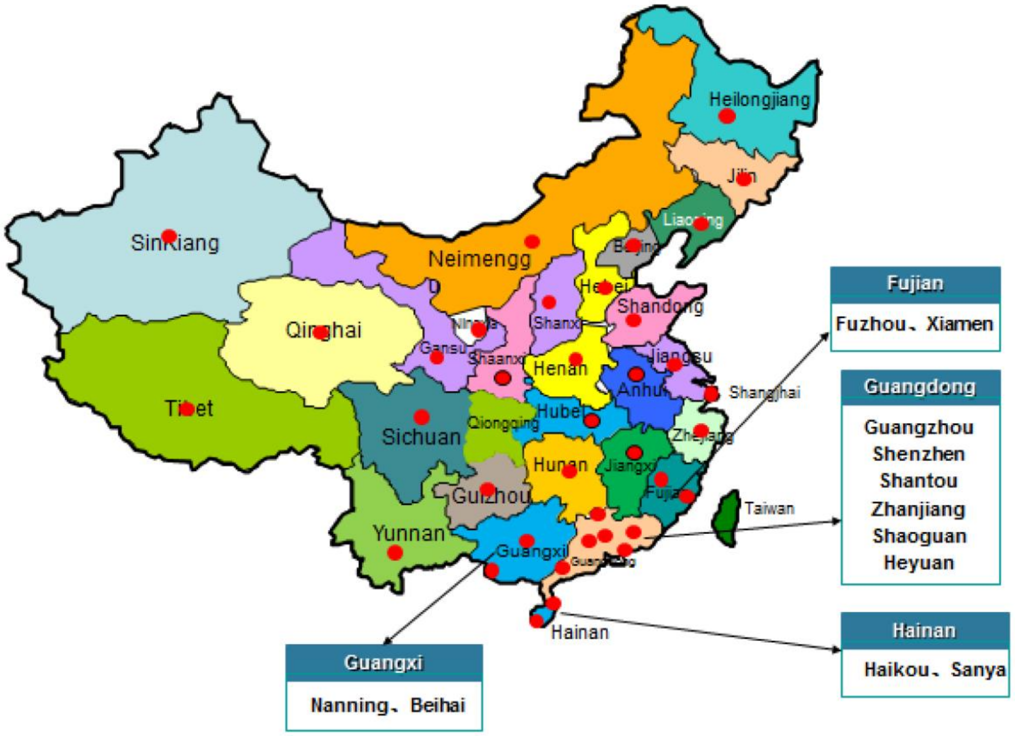

Supplementary Figure 1. Sample collection locations for this study in China.
